# Self-Similarity Simplification Approaches for the Modeling and Analysis of Rockwell Hardness Indentation

**DOI:** 10.6028/jres.107.033

**Published:** 2002-10-01

**Authors:** Li Ma, Jack Zhou, Alan Lau, Samuel Low, Roland deWit

**Affiliations:** National Institute of Standards and Technology, Gaithersburg, MD 20899-8553; Department of Mechanical Engineering and Mechanics Drexel University, Philadelphia, PA 19104; National Institute of Standards and Technology, Gaithersburg, MD 20899-8553

**Keywords:** analytical modeling, cumulative superposition, diamond indenter, FEA, finite element analysis, indentation, Rockwell hardness, self-similarity

## Abstract

The indentation process of pressing a Rockwell diamond indenter into inelastic material has been studied to provide a means for the analysis, simulation and prediction of Rockwell hardness tests. The geometrical characteristics of the spheroconical-shaped Rockwell indenter are discussed and fit to a general function in a self-similar way. The complicated moving boundary problem in Rockwell hardness tests is simplified to an intermediate stationary one for a flat die indenter using principle of similarity and cumulative superposition approach. This method is applied to both strain hardening and strain rate dependent materials. The effects of different material properties and indenter geometries on the indentation depth are discussed.

## 1. Introduction

For a long time, an indentation test, usually referred to as a hardness test, has been the most convenient method for assuring the quality of the mechanical properties of engineering materials. Among the various hardness test methods, the Rockwell hardness (HR) test is the most widely used method for testing metals and other materials due to its simplicity and quickness of execution. In spite of its broad application, Rockwell hardness is not sufficiently standardized at the international level. There are differences in the HR scales of different countries. This could result in technical barriers to global manufacturing and international trade. Since 1997, NIST and other national metrology institutes have been working on the establishment of worldwide-unified Rockwell hardness scales [1]. Among the different Rockwell hardness scales, the Rockwell C hardness (HRC) scale is the most widely used. It employs a diamond indenter, a 98 N (10 kg) preliminary test force and a 1471 N (150 kg) total test force. The HRC value is calculated from the net increase in the penetration depth, as the force on the diamond indenter is increased from the preliminary force to the total force and then returned to the preliminary force. The shape of a Rockwell diamond indenter is conical with a 120° included angle blending tangentially with a spherical tip of 200 μm radius.

There have been many studies that deal with indentation tests using different indenter geometries, such as a spherical indenter (Brinell test), a conical indenter (Cone hardness, HC, O’Neill [2]) and a diamond pyramid indenter (Vickers test, or HV). However, limited studies are found on Rockwell indentation using a spheroconical-shaped diamond indenter. There is an obvious need to increase the understanding of Rockwell hardness tests by correlating results and interpretation with a sound mechanical analysis.

The Rockwell indentation process is conventionally classified into purely elastic, elastoplastic transition and fully plastic regimes. This process is affected by the material’s strain hardening, strain-rate dependency and relaxation. Historically, the development of the indentation field, or contact mechanics, stems from the pioneering research of Heinrich Hertz (1882), which yielded the solution for the frictionless contact of two elastic bodies of ellipsoidal profile. Hertz’s analysis still forms the basis of the design procedures used in many industrial situations involving elastic contact. The Hertz theory is restricted to frictionless surfaces and perfectly elastic solids. Since 1882, the subject of contact mechanics has seen considerable development. Many of the essential results have been summarized [3]. More recent advances contain not only linear elastic contact theories but also include inelastic behavior [4]. Analytical solutions to plastic contact problems are essentially confined to slip line theories of rigid-perfectly plastic solids with simple geometries. Driven by the need to further understand this complicated field of mechanics, a finite element analysis (FEA) method was applied to analyze indentation processes. FEA was first applied to analyze the indentation of elastic-plastic solids under plane and axisymmetric conditions by Hardy et al. [5] and Lee et al. [6], respectively. Since then, FEA has been used by many researchers [7–13] as a general method for contact indentation studies.

The difficulties associated with analyzing indentation problems come from the presence of an unknown and moving contact boundary, nonlinearity and time-dependency. To avoid these difficulties, or at least replace them with more tractable ones, self-similarity was used in the analysis of an intermediate fixed boundary in place of the changing boundary. Linear elastic similarity has been used for a long time. From the late 1980s, self-similarity was applied to nonlinear material properties. Hill et al. [14] utilized self-similarity, in particular, for Brinell indentation of a power-law solid, aided by a specially designed finite element procedure. His investigation was based on a material model with no inherent history dependence, i.e., nonlinear elasticity. The procedure was to use an intermediate flat die field followed by cumulative superposition to analyze indentation of nonlinear solids by curved dies. Using the self-similarity method, indentation of a power law creeping solid was studied by Hill [15], Bower et al. [16] and Storåkers and Larsson [17]. Ogbonna et al. [18] extended it to ball indentation of a transient creep solid. This technique proved to be efficient and was beneficially employed to obtain highly accurate solutions for Brinell indentation and also for strain-hardening plastic solids by Biwa and Storåkers [19]. They later extended the analysis to other shapes. Although the idea is an old one in terms of linear elasticity, apparently the self-similarity approach had never been tried for nonlinear solids where ordinary superposition principles fail to apply.

While complete analysis of fully inelastic behavior is still difficult to achieve by analytical methods, use of a computational method is a powerful tool for obtaining the indentation field when boundary conditions and natural time must be considered simultaneously. After applying FEA to obtain more detailed results, Bower et al. [16], Storåkers et al. [17] and Ogbonna et al. [18] transformed the procedure by Hill [15] to an intermediate rate problem and used the commercial FEA software ABAQUS employing natural time as an essential variable. Biwa and Storåkers [19] and Storåkers et al. [20] also applied ABAQUS to an elastic-plastic procedure.

Although many studies have been made on general geometric indenters, having axisymmetric and non-axisymmetric ball and cone shapes, little research can be found on spheroconical-shaped indenters. Generally, when a cone shape is analyzed, the tip radius is considered zero or so small that it can be ignored. However, experiments have shown that the tip radius of a Rockwell diamond indenter significantly affects the HRC value and cannot be neglected. Ciavarella et al. [21, 22] proposed the solution for a shallow conical indenter with a rounded apex in their study. The indenter was composed of two parts that included the conical and spherical tip that avoided the singularity of the conical tip. His method was still limited to elastic materials. However, for an HRC hardness test, the indentation process includes plastic deformation. To solve this combined spherical and conical plastic indentation problem, the self-similarity method was used here for the Rockwell indenter geometry. The aim is to apply the self-similarity method to the specified Rockwell indenter geometry and offer an efficient solution for describing strain hardening and strain rate-dependent materials. We choose an analytical model for the indenter geometry, which is an approximation to the spheroconical Rockwell indenter but which allows us to use the principle of self-similarity and derive analytical results that closely approximate Rockwell indentation results. This enables us to predict trends and check the results of more detailed finite element analyses.

In Sec. 2, we discuss the profile shape function for a Rockwell diamond indenter. The governing equations and boundary conditions in the indentation process are discussed in Sec. 3. In Sec. 4, the self-similarity simplification approach is analyzed in detail as it is used to obtain displacement, stress and strain distribution information for a Rockwell indentation from flat die indentation results. The analytical results, including the effects that material strain hardening, strain rate dependency, and indenter geometry have on the indentation depth, are discussed in Sec. 5.

## 2. Profile Shape Function for the Rockwell Diamond Indenter

**Fig. 1:**
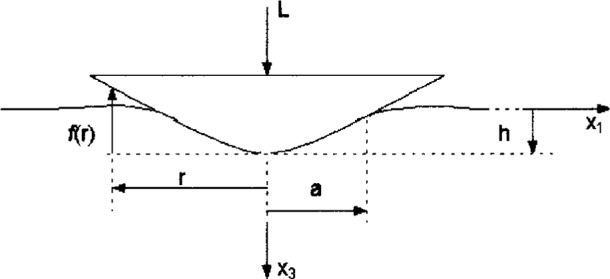
Indenter shape and its indentation of a half-space.

At the beginning of the analysis, it is necessary to know the governing equations and boundary conditions in a strain hardening half-space indentation process (see Fig. 1). In Fig. 1, *x*_1_, *x*_2_ (perpendicular to the drawing surface) and *x*_3_ define a spatial coordinate system, *L* is the applied force, *h* is the indentation depth, and *a* is the maximum contact radius. One key problem in analyzing Rockwell indentation contact is its changing boundary condition. Since the Rockwell indenter is made of diamond and is much harder than the specimen, for simplification, it can be considered as a rigid body.

Referencing Fig. 1, Bower et al. [16] expressed the general curved indenter profile with the following equation,

$$f\left(r\right)=r^{n}/D^{n-1},\;n>0,\;D>0$$ (1)

where $r\left[r={\left(x_{1}^{2}+x_{2}^{2}\right)}^{1/2}\right]$ is the radius of each horizontal cross-section of the indenter*, f*(*r*) is the indenter profile function, and *D* and *n* are indenter geometric constants related to the indenter’s cone angle and curvature. The *f*(*r*) function can be used to model several indenter geometries of practical interest. Its functional form allows us to apply the principle of self-similarity later. Up to now, only ball- and cone-shaped indenters have been discussed. For the cone-shaped indenter, *n* = 1 and *D^n^*^−1^ = tan*β*, where *β* is the cone angle. Alternatively, for the ball-shaped indenter, *n* = 2, and *D* is the diameter of the ball, *D* >> *r*. Hence, Eq. (1) can be used to approximately express the Rockwell indenter profile. To find the suitable values for the constants *n* and *D*, let us first look at the profile of the Rockwell indenter. It can be described by the following two distinct functions:

$$g\left(r\right)=\{\begin{array}{l} 200-\sqrt{200^{2}-r^{2}},\;r\leq 100\;\mu m\\ 200-\sqrt{200^{2}-100^{2}}+\left(r-100\right)\;\tan\;30{}^{\circ},\;r>100\;\mu m \end{array}$$ (2)

**Fig. 2:**
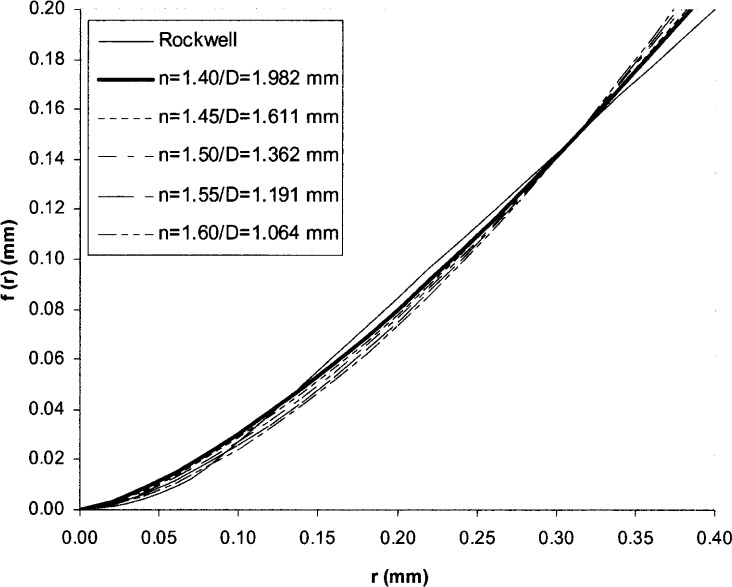
Comparison of estimated profiles and ideal Rockwell indenter profile.

It is obvious that Eq. (2) can only be approximated by Eq. (1). To obtain a suitable approximation, we let the difference between the Rockwell indenter ideal profile (Eq. 2) and estimated profile (Eq. 1) be minimized, then the equation can be written as

$$dq=\frac{1}{R}\int_{0}^{R}\left|g\left(r\right)-f\left(r\right)\right|dr=\min.$$ (3)

where *R* is the maximum contact radius on the indenter profile when testing soft material. For the Rockwell indenter, *n* only can be chosen between 1 and 2. From our previous HRC experiments, it was found that *R* is 400 μm. Integrating *r* from 0 to *R* in Eq. (3), we obtain *n* = 1.4 and *D* = 1982 μm. For these values of *n* and *D*, the difference d*q* between the estimated and the ideal indenter profile is less than 4 %. It was also found that the value of *n* primarily changes the slope of the indenter profile as shown in Fig. 2, affecting both the tip radius and cone angle, while *D* is mainly related to the cone angle.

## 3. Governing Equations in the Indentation Process

For the indentation analysis, it is necessary to know the governing equations and boundary conditions in the indentation process by a Rockwell indenter as illustrated in Fig. 1. It is assumed that the indenter is rigid and pressed normally into the test surface. By imposing indentation depth *h*, the surface boundary *u*_3_ can be described as

$$u_{3}=h-f\left(r\right),\;r\leq a.$$ (4)

The analysis is constrained to a small indentation depth such that the displacements and strains under the indenter can be considered to have a linear relationship. Thus, the small strain tensor **ε***_ij_* is related to the displacement *u_i_* as

$$\varepsilon_{ij}=\frac{1}{2}\left(\frac{\partial u_{i}}{\partial x_{j}}+\frac{\partial u_{j}}{\partial x_{i}}\right),\;\left(i,j=1,2,3\right)$$ (5)

The corresponding strain rate can be expressed as

$${\dot{\varepsilon }}_{ij}=\frac{1}{2}\left(\frac{\partial {\dot{u}}_{i}}{\partial x_{j}}+\frac{\partial {\dot{u}}_{j}}{\partial x_{i}}\right)$$ (6)

where ${\dot{u}}_{i}\left(x_{k}\right)$ is the velocity. The dot denotes differentiation with respect to some monotonically increasing time-like parameter *t*, though not necessarily relying on natural time. Equations (5) and (6) are called compatibility equations.

Generally, a simple uniaxial test is used to determine the material behavior. The equivalent stress *σ*_eq_ is regarded as a unique function of the equivalent strain ***ε***_eq_ and the equivalent strain rate ${\dot{\varepsilon }}_{\text{eq}}$. The material exhibits stain hardening with strain rate-dependent yield strength. The constitutive equation can be expressed in the uniaxial form as

$$\sigma_{eq}=\sigma_{0}\varepsilon_{\text{eq}}^{N}{\dot{\varepsilon }}_{\text{eq}}^{M}$$ (7)

It can be expressed in the more general form as

$$\sigma_{ij}=\sigma_{\text{eq}}\frac{\partial {\dot{\varepsilon }}_{\text{eq}}}{\partial \varepsilon_{ij}}$$ (8)

or

$${\dot{\varepsilon }}_{ij}={\dot{\varepsilon }}_{\text{eq}}\frac{\partial \sigma_{\text{eq}}}{\partial \sigma_{ij}}$$ (9)

where *σ*_eq_ is a function of the stress deviator *S*_i_*_j_* that can be written as

$$S_{ij}=\sigma_{ij}-\frac{1}{3}\sigma_{kk}\delta_{ij}=\sigma_{\text{eq}}\frac{\partial {\dot{\varepsilon }}_{\text{eq}}}{\partial \varepsilon_{ij}}$$ (10)

where the repeated indices of *σ_kk_* are summed.

In the case of both incompressibility and isotropy, the equivalent stress and strain rate are calculated as follows,

$$\sigma_{\text{eq}}=\sqrt{\frac{3}{2}S_{ij}S_{ij}},\;{\dot{\varepsilon }}_{\text{eq}}=\sqrt{\frac{2}{3}{\dot{\varepsilon }}_{ij}{\dot{\varepsilon }}_{ij}},$$ (11)

and

$$\varepsilon_{\text{eq}}=\int {\dot{\varepsilon }}_{\text{eq}}dt$$ (12)

These are of the familiar von Mises type.

The parameters *N* and *M* can be identified as exponents representing strain-hardening and strain rate-sensitivity, respectively. When *N* → 0, the equation represents nonlinear viscous flow or stationary creep, while when *M* → 0, the equation represents strain-hardening plastic flow.

The stress field *σ_ij_* is related to the strain by the above constitutive Eqs. (8) and (9). In the absence of body forces, the stresses must satisfy an equilibrium equation:

$$\frac{\partial \sigma_{ij}}{\partial x_{j}}=0,$$ (13)

for each *i*, where a sum over *j* is assumed. Since a Rockwell indenter is made of diamond, it can be modeled as a rigid body, and the normal displacement imposed by the indenter can be obtained by substituting Eq. (1) into Eq. (4),

$$u_{3}=h-\frac{r^{n}}{D^{n-1}},\;r\leq a.$$ (14)

The boundary conditions between the indenter and the half-space in the frictionless case are

$$\begin{array}{l} {\dot{u}}_{3}=\dot{h},\;\sigma_{13}=\sigma_{23}=0,\;r\leq a\\ \sigma_{13}=\sigma_{23}=\sigma_{33}=0,\;r>a. \end{array}$$ (15)

In addition, we assume that the stresses approach to zero at infinity.

The above equilibrium equation [Eq. (13)], compatibility Eqs. (5) and (6), constitutive Eqs (7), (8) and (9) and boundary condition Eqs. (14) and (15) provide the basis for the solution of the half-space indentation problem.

## 4. Self-Similarity Simplification Approach

The concept of self-similarity was first applied to linear materials for axially symmetric cases by Mossakovski [23] and Spence [24]. The spatial self-similarity of contact for isotropic linear elastic materials was established by Galanov [25, 26] and Borodich [27]. For non-linear power-law materials, it was discovered by Galanov [25] and Borodich [28] in isotropic and anisotropic cases respectively. Later it was applied to creeping materials by Bower et al. [16] and Storåkers et al. [17]. Making use of the concept of self-similarity provided strikingly simple solutions to general problems involving the indentation of a deformable half-space by a rigid body for materials exhibiting power law hardening and strain rate dependent behavior. For Rockwell indentation, the contact area increases with the load. This changing boundary condition combined with nonlinear material behavior, large strain, and time dependence makes the indentation analysis problem complicated for both analytical and computational approaches. However, recent works have shown that indentation of a power-law material by a punch is self-similar, even in the presence of friction [20, 29], so that the complete loading process in such cases can be described by solving a simple flat die problem with fixed boundary conditions. By using the self-similarity method, a transformation of field variables is introduced to convert the changing boundary condition problem to a stationary one that does not depend explicitly on the maximum indentation contact radius, *a*. Hence the Rockwell indentation problem is simplified by solving the problem of a flat die indentation on a strain-rate-dependent material. Therefore, the displacement, strain and stress in the test material can be cumulatively superposed to an intermediate flat die solution. While the cone part of the indenter will introduce large rotations and limit the rigorous applicability of small strain theory and self-similarity, others [16] have used this approach with success and good agreement with experiments. Since the spheroconical indenter is between a cone and a sphere, the rotations will be less and the applicability of the self-similar approach will be even more valid.

The self-similarity principle basically says that for an indenter with a simple shape, like a cone or a flat, the shape of the stress field for a deep indentation is geometrically similar to that for a small indentation. For the transformation, it is assumed the scaled velocities and strain rates are independent of the contact radius *a*. To realize this, Hill et al. [14], Bower et al. [16], Storåkers et al. [17, 20], Biwa and Storåkers [19] considered pure kinematic scaling as

$$r=a\tilde{r}$$ (16)

$$x_{i}=a{\tilde{x}}_{i}$$ (17)

$${\dot{u}}_{i}\left(x_{k},a\right)=\dot{h}{\tilde{u}}_{i}\left({\tilde{x}}_{k}\right)$$ (18)

$$\varepsilon_{ij}\left(x_{k},a\right)=\left(\frac{h}{a}\right){\hat{\varepsilon }}_{ij}\left({\tilde{x}}_{k}\right)$$ (19)

$${\dot{\varepsilon }}_{ij}\left(x_{k},a\right)=\left(\frac{\dot{h}}{a}\right){\tilde{\varepsilon }}_{ij}\left({\tilde{x}}_{k}\right)$$ (20)

$$\sigma_{ij}\left(x_{k},a\right)=\sigma_{0}{\left(\frac{h}{a}\right)}^{N}{\left(\frac{\dot{h}}{a}\right)}^{M}{\tilde{\sigma }}_{ij}\left({\tilde{x}}_{k}\right)$$ (21)

where the scaled variables (~) and (^^^) are independent of the maximum indentation radius *a*. By substituting Eqs. (17), (18), and (20) into Eq. (6), it can be seen that ${\tilde{u}}_{i}$ and ${\tilde{\varepsilon }}_{ij}$ still satisfy the compatibility condition,

$${\tilde{\varepsilon }}_{ij}=\frac{1}{2}\left(\frac{\partial {\tilde{u}}_{i}}{\partial {\tilde{x}}_{j}}+\frac{\partial {\tilde{u}}_{j}}{\partial {\tilde{x}}_{i}}\right)$$ (22)

Then the velocity boundary condition for *r* ≤ *a*, Eq. (15) is normalized by substituting Eq. (18) into Eq. (15) as

$${\tilde{u}}_{3}=1,\;{\tilde{r}}_{i}\leq 1$$ (23)

where ${\tilde{r}}^{2}={\tilde{x}}_{1}^{2}+{\tilde{x}}_{2}^{2}$. This formally corresponds to indentation by a rigid flat indenter with a unit radius.

Similarly by using the above variable substitutions, the field equations can be simplified to:

Equilibrium equation:

$$\frac{\partial {\tilde{\sigma }}_{ij}}{\partial {\tilde{x}}_{j}}=0$$ (24)

Compatibility equation:

$${\tilde{\varepsilon }}_{ij}=\frac{1}{2}\left(\frac{\partial {\tilde{u}}_{i}}{\partial {\tilde{x}}_{j}}+\frac{\partial {\tilde{u}}_{j}}{\partial {\tilde{x}}_{i}}\right)$$ (25)

Constitutive law:

$${\tilde{\varepsilon }}_{ij}={\tilde{\varepsilon }}_{e}\frac{\partial {\tilde{\sigma }}_{e}}{\partial {\tilde{\sigma }}_{ij}},\;{\tilde{\sigma }}_{ij}={\tilde{\sigma }}_{e}\frac{\partial {\tilde{\varepsilon }}_{e}}{\partial {\tilde{\varepsilon }}_{ij}},\;{\tilde{\sigma }}_{e}={\hat{\varepsilon }}_{e}^{N}{\tilde{\varepsilon }}_{e}^{M}$$ (26)

Boundary conditions:

$$\begin{array}{l} {\tilde{u}}_{3}=1,\;{\tilde{\sigma }}_{13}={\tilde{\sigma }}_{23}=0,\;\tilde{r}\leq 1\\ {\tilde{\sigma }}_{13}={\tilde{\sigma }}_{23}={\tilde{\sigma }}_{33}=0,\;\tilde{r}>1 \end{array}$$ (27)

Once the intermediate flat die indentation results are determined, we can superpose the intermediate results to obtain the Rockwell indentation results. Now let us look at how to accomplish the superposition.

By integrating Eq. (18) to equal Eq. (14), we obtain

$$\int_{0}^{1}{\tilde{u}}_{3}\left({\tilde{x}}_{3}\right)\dot{h}dt=h-\frac{r^{n}}{D^{n-1}}$$ (28)

By transforming variable *t* to variable *h* and substituting ${\tilde{x}}_{3}=r/a$, then

$$\int_{0}^{h}{\tilde{u}}_{3}\left(r/a\right)dh=h-\frac{r^{n}}{D^{n-1}},\;r\leq a.$$ (29)

This transformation makes *h* relative to the maximum contact radius *a* and to *r*, and eliminates time *t*. We then rearrange Eq. (29) to

$$h\left(r\right)-\int_{0}^{r}\frac{dh}{ds}{\tilde{u}}_{3}\;\left(r/s\right)ds=h-\frac{r^{n}}{D^{n-1}}.$$ (30)

This is a particular Volterra integral Eq. [30]. Its solution for *h*(*a*) is

$$h\left(a\right)=\frac{a^{n}}{c^{n}D^{n-1}}$$ (31)

where the eigenvalue *c^n^* is defined as

$$c^{n}=1-n\int_{1}^{\infty }\frac{{\tilde{u}}_{3}\left(\tilde{r}\right)}{{\tilde{r}}^{n+1}}d\tilde{r}.$$ (32)

By combining Eq. (31) and Eq (14), the vertical displacement under the indenter is

$$u_{3}=h\left(1-c^{n}\frac{r^{n}}{a^{n}}\right).$$ (33)

From Eq. (33), it can be seen that the vertical displacements at various contact points vary with the indentation depth *h*. At the maximum contact radius *a*, *c^n^* determines the magnitude of the displacement. In particular when *c^n^* < 1, material is pushed below the surface plane at the contact area, which is called “sinking in.” While when *c^n^* > 1, material is piled up at the side of the contact area, which is called “piling-up.”

Next, let us consider the general displacements based on the integral of Eq. (18), which serves to superpose the unit flat die indentations,

$$u_{i}\;\left(x_{k},a\right)=\int_{0}^{a}\frac{dh}{ds}{\tilde{u}}_{i}\;\left(x_{k}/a\right)ds.$$ (34)

By a series of mathematical operations of derivation, substitution and variable transformation, and by referring to Eq. (31), Eq. (34) can be written as:

$$u_{i}\;\left(x_{k},a\right)=nh{\left(r/a\right)}^{n}\int_{\tilde{r}}^{\infty }\frac{{\tilde{u}}_{i}\left({\tilde{x}}_{k}\right)dh}{{\tilde{r}}^{n+1}}\;d\tilde{r}.$$ (35)

The equation for obtaining strain is derived in the same way as that for obtaining displacement,

$$\varepsilon_{ij}\;\left(x_{k},a\right)=n\frac{h}{a}{\left(\frac{r}{a}\right)}^{n-1}\int_{\tilde{r}}^{\infty }\frac{{\tilde{\varepsilon }}_{ij}\left({\tilde{x}}_{k}\right)}{{\tilde{r}}^{n}}\;d\tilde{r}.$$ (36)

To obtain **ε***_ij_*, it can be seen that it is necessary to integrate a generic point from *x_i_*/*a* to infinity.

In the Rockwell indentation test, the main concern is the dependence of the resulting indentation depth on the applied force. The total applied force *L* can be obtained by multiplying *σ*_33_ in Eq. (21) by differential the scaled contact area *Ã* and integrating as

$$L=a^{2}\sigma_{0}{\left(\frac{h}{a}\right)}^{N}{\left(\frac{\dot{h}}{a}\right)}^{M}\int \left(-{\tilde{\sigma }}_{33}\right)d\tilde{A}.$$ (37)

where the scaled contact area *Ã* is related to the real contact area *A* as:

$$A=a^{2}\tilde{A}.$$ (38)

Substituting Eq. (31) into Eq. (37), we now express the applied force *L* in terms of the indentation depth *h* as:

$$L=c^{2}h^{2/n}D^{2\left(n-1\right)/n}\sigma_{0}{\left(\frac{h^{\left(n-1\right)/n}}{D^{\left(n-1\right)/n}c}\right)}^{N}{\left(\frac{\dot{h}}{ch^{1/n}D^{\left(n-1\right)/n}}\right)}^{M}\int \left(-{\tilde{\sigma }}_{33}\right)d\tilde{A}.$$ (39)

This equation is crucial for determining the effects of the applied force and indenter geometries on the indentation depth. So we have described in detail how a complicated Rockwell indentation problem, involving both a moving boundary condition and time dependence, can be transformed to a stationary flat die problem.

## 5. Analytical Results and Discussion

To obtain the Rockwell indentation analytical results, the first step is to obtain the analytical results for an intermediate Rockwell indentation problem based on the equations derived above, which correspond to a unit flat die indentation solution. Bower et al. [16] give the solution for *M* = 1, which is the linear viscous solid material, using the analytical method. For other cases, the solution to the simplified flat die indentation problem has to be calculated using a finite element method. FEA models for the flat die indentation problem have been analyzed in detail by Bower et al. [16] for a power law creep material, by Ogbonna et al. [18] for a rate-independent and rate-dependent material, by Biwa and Storåkers [19] for a plastic flow, by Storåkers and Larrson [17] for stationary creep, and by Storåkers et al. [20] for rate independent strain-hardening materials and power law viscous materials. All of them have utilized the flat die indentation approach to solve the ball indentation problem. The flat die indentation solution involves a singularity at the contact boundary similar to an elasto-plastic crack tip field. Nevertheless, the self-similarity approach has been shown to be applicable to different indenter geometries to determine most of the deformation parameters, such as the contact boundary, indentation depth and indentation load [15–20].

Based on the flat die indentation FEA results, we can derive the Rockwell indentation analytical results. Unlike the Brinell hardness test, which is mainly concerned with the average pressure under the indenter and maximum contact radius *a*, Rockwell hardness is determined by the indentation load, relative depth, and indenter geometry. From Eq. (39), the indentation depth relation can be calculated as follows,

$$h\frac{2+nN-\left(M+N\right)}{n}=\frac{L}{\sigma_{0}c^{2-N-M}D^{\left(n-1\right)\left(2-M-N\right)/}{\dot{h}}^{M}\tilde{L}}$$ (40)

where $\tilde{L}$ corresponds to the dimensionless force applied to the flat die indenter and is defined by the equation,

$$\tilde{L}=\int \left(-{\tilde{\sigma }}_{33}\right)d\tilde{A},$$ (41)

and $\tilde{L}$ can be obtained from the FEA results of a flat die indentation. The constant *c* for different material and indenter geometries can be obtained from Eq. (32). It denotes the transform factor from the flat die indenter to the Rockwell indenter. The force factor $\tilde{L}$ is only related to the material parameters *M* and *N*. Since *c* and $\tilde{L}$ for the intermediate flat die indentation are constants for individual material, we shall use *c* and $\tilde{L}$ from the FEA result of Bower et al. [16] for the following parameter relationship analysis and discussion. The constant *c* for *n* = 1.4 is obtained from a linear interpolation of *c* between *n* = 1 and n = 2. Based on plastic flow theory, *c*(*M*) is very close to *c*(*N*) as compared to the creep results [19,20], so *c*(*M*) is set equal to *c*(*N*) for the same *M* and *N* value in our following analysis.

### 5.1 Indentation Force and Depth Relation for Different Materials

In Rockwell hardness indentation, the indentation depth in reaction to certain indentation forces is of primary importance. By substituting the constants *c* and $\tilde{L}$ into Eq. (40), the relationships for strain rate independent and strain rate dependent materials are found as follows.

#### 5.1.1 Strain Rate Independent Material

**Fig. 3:**
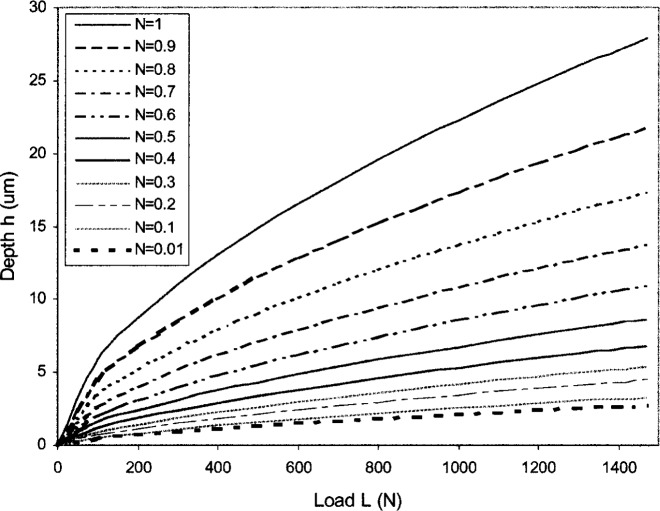
Indentation depth for different strain hardening material.

When the strain rate dependence exponent *M* is 0, the material is strain rate independent, and the constitutive Eq. (7) is reduced to

$$\sigma_{\text{eq}}=\sigma_{0}\;\varepsilon_{\text{eq}}^{N},\;\left(0<N<1\right)$$ (42)

which gives the power law response of a strain hardening plastic solid. By using Eq. (40), the load-depth relationship for a strain hardening material of *N* from 0.01 to 1 is derived and shown in Fig. 3.

It can be seen that the indentation depth increases with the increasing indentation force for the each strain hardening material. As the strain hardening factor *N* increases, i.e., the testing block material hardens less strongly with strain, the indentation depth also increases under the same applied force.

#### 5.1.2 Strain Rate Dependent Material

**Fig. 4:**
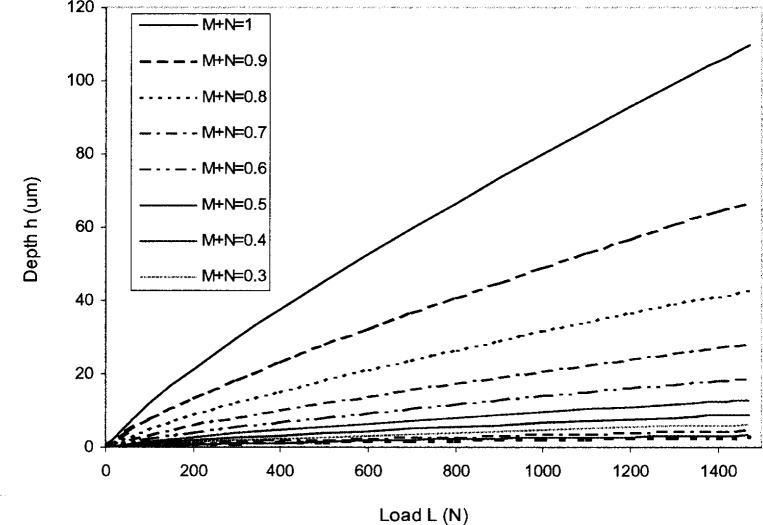
Indentation force-depth relation for various *M* + *N* values, i.e., for different strain rate dependent material.

When the strain rate dependence exponent *M* is different than 0, the material is strain rate dependent. When considering both strain hardening and strain rate effects, for the same value of *M* and *N*, *c*(*M*) and *c*(*N*) are almost identical [19, 20]. We let *c*(*M*) = *c*(*N*) for the same *M* and *N* values, as the sum of *M* and *N* increases from 0.01 to 1.0. When substituting c(*M*) and *c*(*N*) into Eq. (40), the resulting applied force-depth relation for strain rate dependent material with strain hardening is shown in Fig. 4. It can be seen that indentation depth increases with increasing indentation force for each strain rate dependent material. When both the strain rate dependent exponent *M* and the strain hardening factor *N* increase, the material hardens less quickly with strain and strain rate, and under the same applied force, the indentation depth increases.

### 5.2 Effect of Rockwell Indenter Geometry Parameter *D* on Indentation Depth

**Fig. 5:**
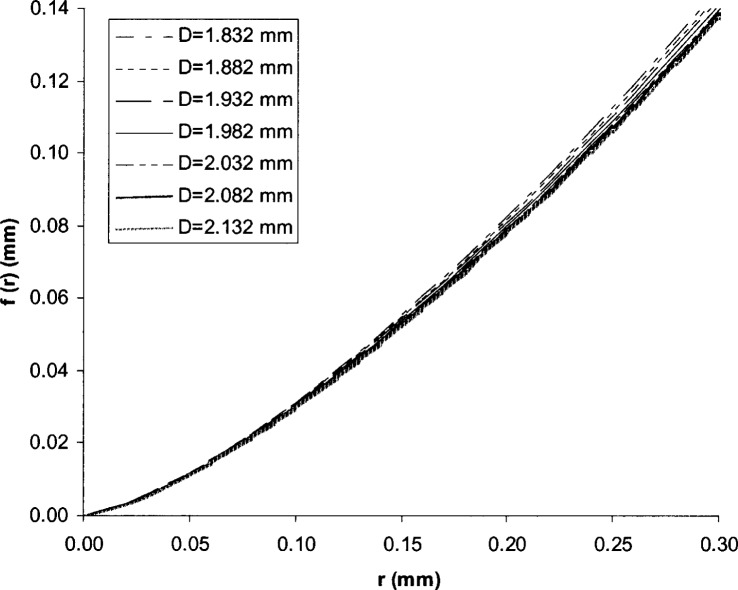
The effect of *D* on Rockwell indenter geometry.

**Fig. 6:**
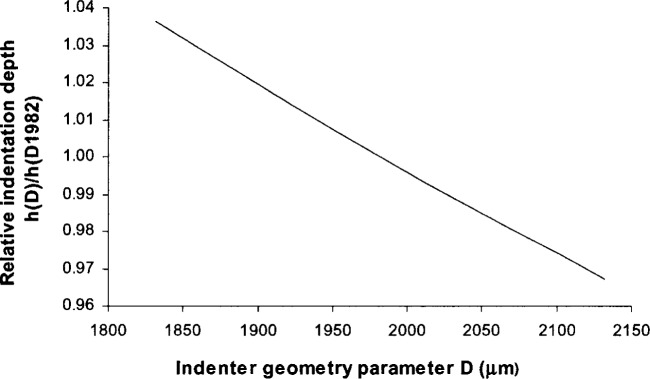
The effect of the indenter cone angle parameter *D* on the indentation depth from analytical results.

In a Rockwell hardness test, the indenter’s cone angle affects the HRC value. From the indenter geometry equation Eq. (1), the Rockwell indenter profile can be plotted with changes in parameter *D* (Fig. 5). From Fig. 5, it can be seen that *D* mainly affects the indenter cone angle, since all of the curves exhibit obvious differences only beyond the 100 μm range of the spherical tip of the indenter. The Rockwell indenter cone angle increases as parameter *D* increases. Substituting different values of the parameter *D* into Eq. (40), we can predict the trend of cone angle effect under the same applied force, same material, and the same indentation speed (see Fig. 6). In Fig. 6, the indenter geometry parameter *n* was chosen at the reference value of 1.4, and *D* was varied around the reference value of 1982 μm. From Fig. 6, it can be seen that when D, or the cone angle increases, the indentation depth decreases. This trend agrees with Barbato’s experimental results [31, 32].

### 5.3 Effect of Rockwell Indenter Geometry Parameter *n* on Indentation Depth

**Fig. 7:**
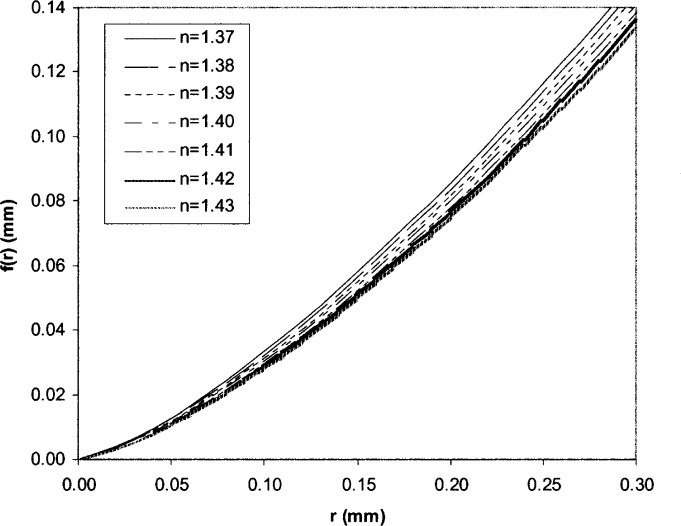
The effect of *n* on Rockwell indenter geometry.

**Fig. 8:**
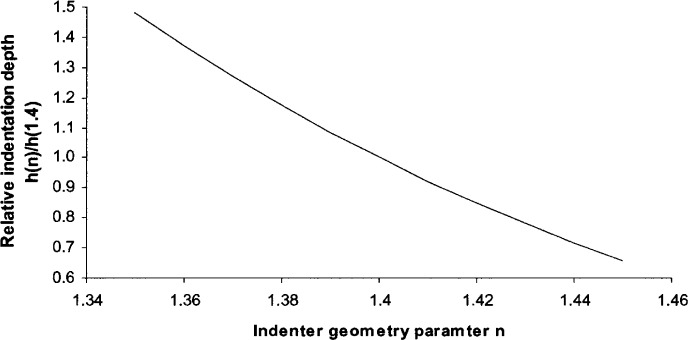
The effect of the indentation geometry parameter *n* on the indentation depth from analytical results.

To check the effect of the parameter *n*, we plotted the Rockwell indenter profile with the changes in *n* (see Fig. 7). From Fig. 7, it can be seen that both tip radius and cone angle increase when the parameter *n* increases. From Eq. (40), when applying the same force to the same material under the same indentation speed, the indentation depth decreases with *n* (see Fig. 8). In Fig. 8, the indenter geometry parameter *D* is kept as the reference value of 1982 μm, only *n* is changed. It can be seen that when *n*, or both the cone angle and tip radius increase, the indentation depth decreases. This trend is also in agreement with Barbato’s experimental results [31, 32].

## 6. Conclusion

The Rockwell indentation process has been analyzed and modeled for both strain hardening and strain rate dependent materials. To simplify the Rockwell indenter’s spheroconical shape, it was fit to a general function with a self-similarity property, which simplified the problems of complicated changing boundary conditions and nonlinear material properties. It was demonstrated that by principles of similarity and cumulative superposition, the complicated moving boundary problem could be simplified to an intermediate stationary one for a flat indenter. The effects of material strain hardening, strain rate parameters, and indenter geometry parameters on the indentation depth were analyzed. From the analytical results, it can be seen that indentation depth decreases as the indenter geometry parameters *D* or *n* (which correspond to the cone angle or both the tip radius and cone angle, respectively) increase.

## References

[1] Song JF, Low S, Pitchure D (1997). Establishing a world-wide unified Rockwell hardness scale with metrological traceability. Metrologia.

[2] O’Neill H (1967). Hardness Measurement of Metals and Alloys.

[3] Hills DA, Nowell D, Sackfield A (1993). Mechanics of elastic contacts.

[4] Johnson KL (1987). Contact Mechanics.

[5] Hardy C, Baronet CN, Tordion GV (1971). The elasto-plastic indentation of a half-space by a rigid sphere. Internatl J Numer Meth Eng.

[6] Lee CH, Masaki S, Kobayashi S (1972). Analysis of ball indentation. Internatl J Mech Sci.

[7] Edlinger ML, Gratacos P, Montmitonnet P, Felder E (1993). Finite element analysis of elastoplastic indentation with a deformable indenter. Europe J Mech Solids.

[8] Kral ER, Komvopoulos K, Bogy DB (1993). Elastic-plastic finite element analysis of repeated indentation of a half-space by a rigid sphere. J Appl Mech, Trans ASME.

[9] Care G, Fischer-Cripps AC (1997). Elastic-plastic indentation stress fields using the finite-element method. J Mater Sci.

[10] Fischer-Cripps AC (1997). Elastic-plastic behaviour in materials loaded with a spherical indenter. J Mater Sci.

[11] Bhattacharya AK, Nix WD (1988). Finite element simulation of indentation experiments. Internatl J Solids and Struct.

[12] Faulkner A, Tang KC, Sen S, Arnell RD (1998). Finite element solutions comparing the normal contact of an elastic-plastic layered medium under loading by (a) a rigid and (b) a deformable indenter. J Strain Anal.

[13] Sen S, Aksakal B, Ozel A (1998). A finite-element analysis of the indentation of an elastic-work hardening layered half-space by an elastic sphere. Internatl J Mech Sci.

[14] Hill R, Storåkers B, Zdunek AB (1989). A theoretical study of the Brinell hardness test. Proc R Soc Lond A.

[15] Hill R (1992). Similarity analysis of creep indentation tests. Proc R Soc Lond.

[16] Bower AF, Fleck NA, Needleman A, Ogbonna N (1993). Indentation of a power law creeping solid. Proc R Soc Lond.

[17] Storåkers B, Larsson P-L (1994). On Brinell and Boussinesq indentation of creeping solids. J Mech Phys Solids.

[18] Ogbonna N, Fleck NA, Cocks ACF (1995). Transient creep analysis of ball indentation. Internatl J Mech Sci.

[19] Biwa S, Storåkers B (1995). An analysis of fully plastic Brinell indentation. J Mech Phys Solids.

[20] Storåkers B, Biwa S, Larsson PL (1997). Similarity analysis of inelastic contact. Internatl J Solids Struct.

[21] Ciavarella M, Hills DA, Monno G (1998). Contact problems for a wedge with rounded apex. Internatl J Mech Sci.

[22] Ciavarella M (1999). Indentation by nominally flat or conical indenters with rounded corners. Internatl J Solids Struct.

[23] Mossakovski VI (1963). Compression of elastic bodies under condition of adhesion (axisymmetric case). Prik Mat Mekh.

[24] Spence DA (1968). Self similar solutions to adhesive contact problems with incremental loading. Proc R Soc, London A.

[25] Galanov BA (1981). Approximate solution to some problems of elastic contact of two bodies. Mech Solids.

[26] Galanov BA (1981). Approximate solution of some contact problems with an unknown contact area under conditions of power law of material hardening. Dokl AN Ukrainia SSR, Part A.

[27] Borodich FM (1983). Similarity in the problem of contact between elastic bodies. J Appl Math Mech.

[28] Borodich FM (1989). Hertz contact problems for an anisotropic physically nonlinear elastic medium. Strength Mater.

[29] Borodich FM (1993). The Hertz frictional contact between nonlinear elastic anisotropic bodies (the similarity approach). Internatl J Solids Struct.

[30] Zwillinger D (1998). Handbooks of Differential Equations.

[31] Barbato G, Desogus S, Germak A (1998). Experimental analysis on the influence quantities in the Rockwell C hardness test.

[32] Barbato G, Galetto M (1998). Influence of the indenter shape in Rockwell hardness test.

